# Talin Is Required Continuously for Cardiomyocyte Remodeling during Heart Growth in *Drosophila*

**DOI:** 10.1371/journal.pone.0131238

**Published:** 2015-06-25

**Authors:** Simina Bogatan, Duygu Cevik, Valentin Demidov, Jessica Vanderploeg, Abdullah Panchbhaya, Alex Vitkin, J. Roger Jacobs

**Affiliations:** 1 Department of Biology, McMaster University, Hamilton, ON, Canada; 2 Department of Medical Biophysics, University of Toronto, Toronto, ON, Canada; 3 Department of Biology, Taylor University, Euler Science Complex, 236 W. Reade Ave, Upland, IN, 46989, United States of America; Heart Science Centre, Imperial College London, UNITED KINGDOM

## Abstract

Mechanotransduction of tension can govern the remodeling of cardiomyocytes during growth or cardiomyopathy. Tension is signaled through the integrin adhesion complexes found at muscle insertions and costameres but the relative importance of signalling during cardiomyocyte growth versus remodelling has not been assessed. Employing the *Drosophila* cardiomyocyte as a genetically amenable model, we depleted the levels of Talin, a central component of the integrin adhesion complex, at different stages of heart growth and remodeling. We demonstrate a continuous requirement for Talin during heart growth to maintain the one-to-one apposition of myofibril ends between cardiomyocytes. Retracted myofibrils cannot regenerate appositions to adjacent cells after restoration of normal Talin expression, and the resulting deficit reduces heart contraction and lifespan. Reduction of Talin during heart remodeling after hatching or during metamorphosis results in pervasive degeneration of cell contacts, myofibril length and number, for which restored Talin expression is insufficient for regeneration. Resultant dilated cardiomyopathy results in a fibrillating heart with poor rhythmicity. Cardiomyocytes have poor capacity to regenerate deficits in myofibril orientation and insertion, despite an ongoing capacity to remodel integrin based adhesions.

## Introduction

The heart has remarkable capacity to respond to changes in hemodynamic load during growth and aging of the cardiovascular system. Changes in vertebrate heart structure and physiology result in part from the addition of new cells, and the remodelling of existing myocytes. As the heart grows, cardiomyocytes increase the length of their myofibrils. Concentric hypertrophic remodelling of the heart increases force by adding myofibrils in parallel. Dilated cardiomyopathy, resulting from sustained overload, is reflected in a decrease in myofibril length and number [1, 2]. These structural changes in myocytes are triggered by mechanotransduction of tension. Although there may be multiple signalling pathways recruited, the primary path for tension signalling to the cytoskeleton is through its links to the extracellular matrix (ECM) by integrins—at the intercalated disc and the costameres. Intercalated discs are the end-to end junctions between cardiomyocytes that transmit mechanical tension across heart muscle, and sense heart load. Costameres are Integrin and Dystroglycan based linkage between the muscle Z-line and the ECM. **β-1** integrin knockout mice exhibit reduced cardiac tolerance to increased hemodynamic load [3]. Integrin second messengers, such as focal adhesion kinase and integrin linked kinase are postulated to mediate tension signalling [4, 5]. Expression of Integrins and proteins of the Integrin Adhesion complex (IAC) are altered during heart remodelling [6–8], and mutations in Integrin ligands or the IAC are associated with human cardiomyopathies [9–11].

In this report we examine the role of the IAC component Talin in remodelling of cardiomyocytes. The Talin dimer acts as a physical link between the Integrin dimer and the actin cytoskeleton, as well as Vinculin and Focal Adhesion kinase [12, 13]. Talin’s engagement of actin is modulated by Integrin-ECM adhesion, and Talin can reciprocally alter Integrin- ligand affinity. Mammals have two Talin genes that are differentially localised to cardiac muscle intercalated discs and to the costamere [14]. Up-regulation of Talin 1 in the hearts of Fragile X autosomal homolog1 mutants is associated with disruption of costamere structure in heart muscle [15]. Increased cardiac load will increase Talin1 expression at costameres. Knock-out of Talin1 in the adult mouse heart generates no structural defects, perhaps because Talin2 is the major form expressed after differentiation. Nevertheless, the hypertrophic responses to increased load are reduced in the absence of Talin1 [16]. These studies suggest that the IAC mediates dynamic responses to tension in cardiomyocytes, ideally revealed by temporally manipulating protein function during growth and changes in hemodynamic load.

Significant insights into cardiomyopathies at the genetic and molecular level have been generated by study of genetic model organisms, such as Zebrafish and *Drosophila melanogaster* [17, 18]. The *Drosophila* dorsal vessel consists of an open tube, with a posterior heart and anterior aorta in which spontaneous rhythmicity is generated by posterior myocytes [19]. The development and function of the *Drosophila* heart shows remarkable molecular conservation with vertebrates [20, 21] and has been instructive in revealing the mechanisms of cardiomyopathy [22, 23]. Unlike the mammalian heart, the *Drosophila* heart has no stem cells, and no new cardiomyocytes are generated during growth or aging. Nevertheless, the heart grows 5 fold in length from embryo to adulthood (our observations), and undergoes significant shape remodelling during metamorphosis [24]. Therefore, this model provides a unique *in vivo* and genetic model of the remodelling capacity of individual cardiomyocytes.

In this study, we examine the role of Integrin adhesion and signalling in growth and remodelling of the *Drosophila* heart. The cytoplasmic linker, Talin, is our focus, because *Drosophila* has a single talin gene (*rhea*) which is required for both Integrin adhesion and signalling [12, 25]. For the first time in a genetic model, we have transiently depleted the levels of an integrin adhesion complex protein at several different stages of growth and morphogenesis of the heart.

Temporally restricted depletion of Talin by cardiomyocyte directed expression of dsRNA reveals differential dependence upon Talin turnover in the IAC during heart growth and heart remodelling. Reduced cardiomyocyte Talin results in cell retraction from cardiomyocyte cell junctions, which are functionally similar to the intercalated disc, resulting in fewer and shorter myofibrils. Remarkably, restoration of Talin expression does not enable regrowth or regeneration of cell insertions, so that early damage persists through the life of the cardiomyocyte. These data indicate that cardiomyocytes require ongoing Talin function for maintenance and growth of adhesions, and Talin expression is not sufficient for myocyte regeneration.

## Methods and Materials

### Genetics

*HandGal4* was provided by A. Paululat [26], and *tubGal80* ^*ts*^ from the Bloomington Stock Centre (Bloomington, Indiana, USA). Tin-CΔ4 Gal4 was not used because of its low expression at larval stages. Both *UAS-dsβ1-integrin RNA* (103704) and *UAS-dsTalin RNA* (40399) have been previously validated [27] (see also S1 File), and were obtained from the Vienna *Drosophila* Resource Centre (Vienna, Austria). Our own Talin RNAi results were further corroborated with *TRiP*.*HM05161}attP2* (Bloomington 28950). Both *rhea* (Talin) double stranded sequences target all four predicted ORFs, which are not yet assessed for tissue specific transcripts [28]. Flies were raised at temperatures permissive (29°C) and non-permissive (18°C) for Gal4 to regulate the timecourse of Talin dsRNAi expression. The limited perdurance of dsRNAi after temperature shift is demonstrated in S1 File. First larval instar where shifted within 6 hrs of hatching, for 24 hrs. The second instar shift began after 48 hrs at 18°C, for a duration of 30 hours. The third instar shift occurred after 96 hours at 18°C. Pupal shifts occurred when wandering larvae emerged from the food, and the adult shift occurred within 4 hours of eclosion.

### Immunolabeling

Adult *Drosophila melanogaster* females were dissected at either 6–8 days or 42–46 days after eclosion in artificial hemolymph and fixed in 4% paraformaldehyde (Polysciences, Warrington, PA, USA) [29]. After washing and blocking, dissections were incubated overnight at 4°C in antibody. β-integrin, Talin and Pericardin antibodies were obtained from the Developmental Studies Hybridoma Bank, Iowa City. IA, USA. Zasp antibody was the gift of Frieder Schöck [30]. Secondary Alexa 647 anti-mouse (1:200) and Alexa 546 conjugated phalloidin (1:200) (LifeTechnologies, Burlington ON, Canada) were used to visualize labeling with a Leica TCS SP5 confocal microscope. Confocal images are projections, subsequent to equivalent adjustment of brightness for each panel.

### Electron microscopy

Dissections were fixed in 4% paraformaldehyde and 2.5% glutaraldehyde in cacodylate buffer, post-fixed in 1% osmium tetroxide, and stained in uranyl acetate before embedding in Epon-Araldite (Polysciences, Warrington, PA, USA). Lead stained 0.1 μm sections were examined on a JEOL 1200EXII microscope at 80 kV. We examined over 80 sections from 4 specimens of each genotype.

### Longevity

Adult males collected within 4 hours of eclosion were aged 10/vial at 18 or 29°C, and provided fresh vials every 5 days.

### Optical coherence tomography

OCT is based on low-coherence interferometry to enable micron-scale non-invasive tomographic imaging in biological tissues to a depth of 1–3 μm. The OCT system used here has been described previously [31]. Briefly, images were acquired with a 36 kHz (depth scan rate) Fourier domain mode-locked OCT system utilizing a swept laser source based on a tunable polygon filter configuration with a sweeping range of 110 nm centered at 1310 nm. This yields axial and lateral resolutions of 8 μm and 13 μm in tissue, respectively.

M-mode records of larval heart contraction were analysed for rhythmicity index (RI) and heart rate, as shown (see S2 File). Larval heart dilation and diastolic/systolic ratios were measured from 20 cycles for each heart in segment A6 with ImageJ. The 20 cycle fragments were selected for a lack of interference from body or gut peristalsis, which will arrest the heartbeat. As quiescent periods were under-sampled by this approach, we opted to analyse heart rhythm with an autocorrelation approach used in earlier Drosophila heart studies [32, 33], rather than by the arrhythmia index [34]. The Rhythmicity Index (RI), a measure of rhythm strength, is a correlation coefficient, generated employing the approach of Levine [35], in which the height of the third peak of an autocorrelation plot is taken as the RI. Each treatment was examined for 6–20 animals. All data sets were analysed with Mann Whitney U tests for significance and presented with the Standard Error of the Mean (s.e.m.).

## Results

**Fig 1:**
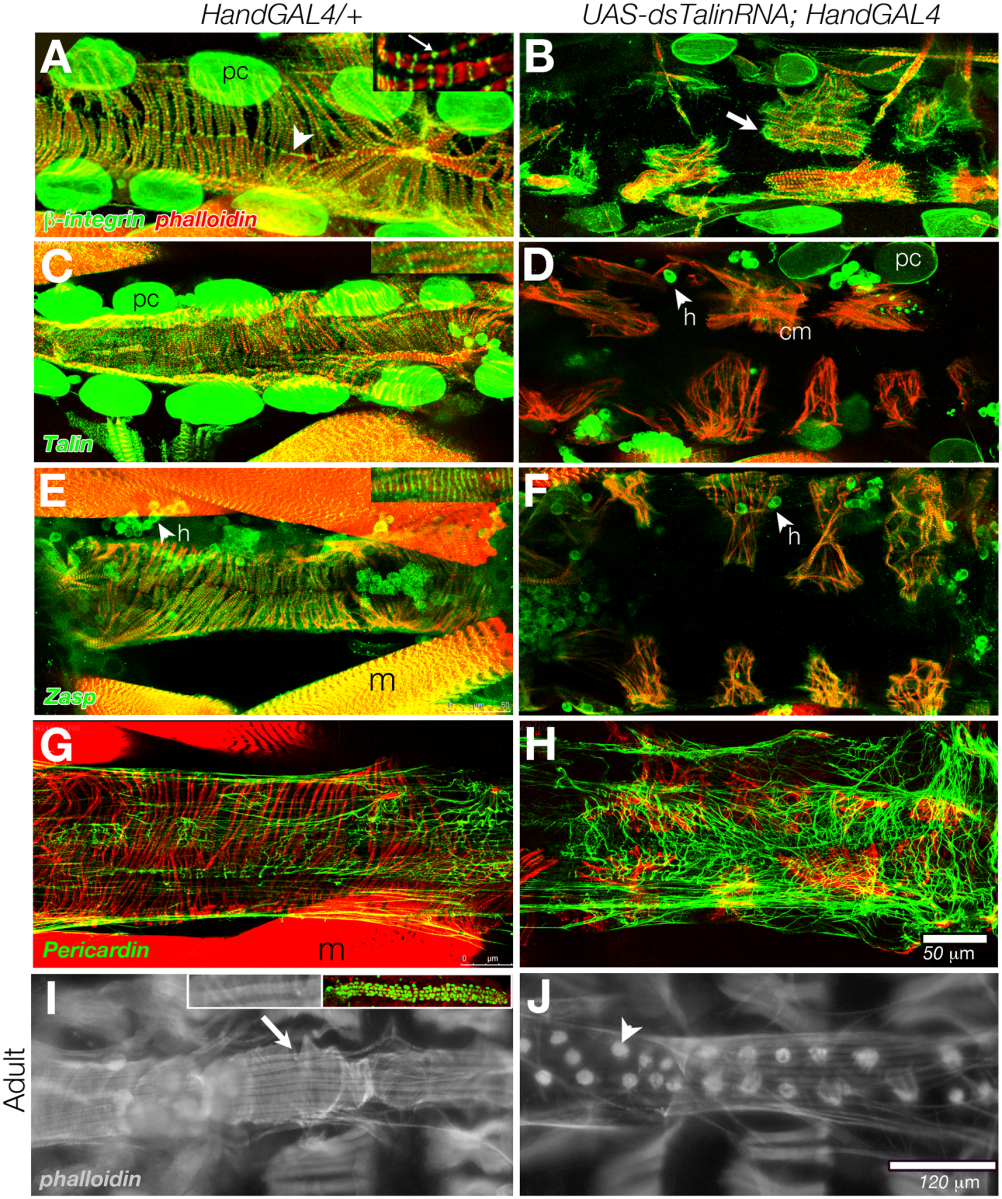
Talin depletion in cardiomyocytes resulted in retraction of the muscle actin network. The third instar larval hearts lack longitudinal fibres, revealing the transverse fibres (phalloidin, red) and integrin rich cell insertions at the midline. Confocal projections of abdominal segments 5 and 6 of dissected, immunolabled control (Hand-GAL4/+) hearts are shown (arrowhead, β-integrin, green, in A). The contractile heart is flanked by integrin rich pericardial cells (pc). When imaging single myofibrils, Integrin antibody also labeled myocyte Z-lines (arrow in inset, A). When dsTalinRNA expression was generated in UAS-*dsTalinRNAi (Hm0516); Hand-Gal4* and examined in third instar larvae, partial myofibril retraction was observed (B). Integrin was concentrated at the cell periphery, beyond the limit of myofibril projection (arrow, B). Talin distribution in wildtype is similar to β-integrin (C), but after chronic depletion in the heart, levels are low in pericardial cells (pc) and cardiomyocytes (cm) but still abundant in hemocytes (h) (D). Zasp, a component of the costamere, aligns to heart and body wall muscle Z-lines, and in hemocytes (E, and inset). This pattern is maintained in myofibrils subsequent to Talin depletion (F). The larval heart ECM contains a latticework of Pericardin rich fibrils (G). After Talin depletion, this latticework becomes more dense in the pericardium (H). The myocytes of the adult control (Hand-Gal4/+) heart are comprised of longitudinal fibres (arrow) overlying the transverse fibres of the cardiomyocytes (I, phalloidin labeling). Relative to the embryonic heart at hatching (inset I, labeled with Hand-GFP), the adult heart has grown 4.7 times in length. dsTalinRNA expression was generated in UAS-*dsTalinRNA; HandGAL4 (VDRC 40399)* larvae, generating a more severe phenotype, resulting in adult escapers with a collapsed myofibril network, where actin bundles surround each myocyte nucleus (arrowhead, J). Posterior is at right. Scale: 50 μm in A-H; 120 μm in I,and J.

*Drosophila* have an open circulatory system, in which the contractile heart and aorta comprise a tube with an anterior opening. The larval and adult hearts have a single layer of cardiomyocytes that enwrap the posterior heart chamber and the anterior aorta, with myocytes in close apposition to their contralateral partners at the dorsal and ventral midlines [36, 37]. *Drosophila* hearts express a single β-integrin, βPS, encoded by *myospheroid [38, 39]*. We examined the distribution of βPS immunolabeling in the cardiomyocytes of actin (phalloidin) labeled dissected larval and adult hearts (Fig 1A and 1I). Myofibrils (discrete bundles of actomyosin filaments) were uniformly distributed around the vessel, and each myofibril was matched to a contralateral myofibril across an integrin rich cell junction, analogous to an intercalated disc (arrowhead, Fig 1A). In addition, integrin was enriched on the myocyte surface at each costamere, revealed in a striated pattern of both actin and integrin labeling (Fig 1A, inset). The heart was flanked by pairs of integrin-rich pericardial cells, which function as nephrocytes. After metamorphosis, the adult heart has an additional layer of longitudinal fibres that overlies the cardiomyocytes (Fig 1I).

### Talin is required to maintain cardiomyocyte insertions

Integrin or Talin levels can be selectively reduced in the cardiomyocytes by dHAND-Gal4 directed expression of dsRNA. Reduction of integrin levels by targeted expression of dsRNA for βPS caused complete retraction of all myofibrils, and an accumulation of actin around the nucleus of each cardiomyocyte ([40]; our unpublished observations). Continuous depletion of Talin during larval stages in the heart resulted in pupal death for 85% (n = 55) of flies. We immunolabeled ‘escaper’ adults and verified that low levels of dsRNA expression for Talin generated the same effect as βPS depletion, consistent with the Talin linker protein mediating integrin function in stabilising myofibrils (Fig 1J). Less complete myofibril retraction resulted when Talin levels were reduced transiently during larval growth, and assessed in the third instar. Myocytes appeared to have lost contact with their ipsilateral and contralateral partners, and integrin labeling increased at the cell periphery (Fig 1B). The heart also appeared to be wider. This less severe phenotype suggests that the requirement for Talin may change during growth and metamorphosis of the heart, which is explored further below. We also examined the effects of Talin depletion on proteins of the costamere complex. Integrin, Talin and Zasp localise to the costameres of cardiomyocytes of control larval hearts (Fig 1A, 1C and 1E). Subsequent to Talin depletion, a low level of disorganised Talin immunolabel was detected in cardiomyocytes (*cm* in Fig 1D). Integrin and Zasp was still detected at costameres- although Zasp levels were visibly lower (Fig 1B, 1F). In contrast to the myocyte retraction phenotype, costamere structure is less sensitive to the level of Talin expression. We also examined the organisation of the heart ECM. The heart is surrounded by a meshwork of Pericardin rich fibrils (Fig 1G). Pericardin is a collagen-like protein characteristic of the abluminal heart ECM [41]. The ECM meshwork was more elaborate in Talin depleted larval hearts, perhaps in response to the widening of the heart (Fig 1H).

### Reduced larval Talin expression results in heart dilation

**Fig 2:**
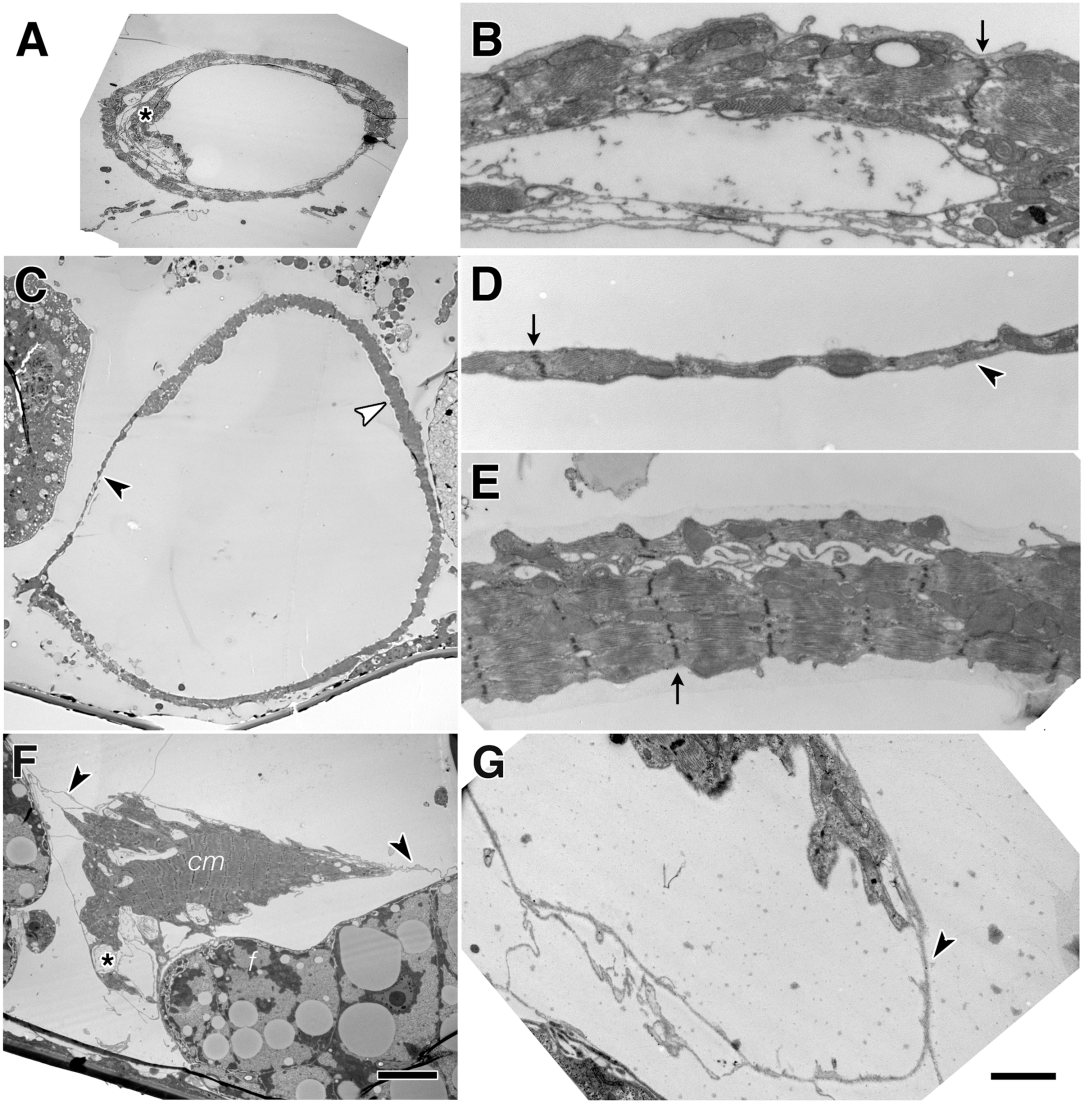
Ultrastructure of Talin depleted larval hearts reveal dilation of the lumen. The wildtype LIII dorsal aorta (segment A4) was completely encompassed with myofibrils (A, B). Arrows mark Z lines of myofibrils. Extensions of a valve cell line the left and ventral lumen (asterisk, A). After continuous Talin depletion in UAS-dsTalinRNA; Hand-GAL4, the aorta lumen was dilated (C). The aorta wall had zones with myofibril (white arrowhead, C and E) and without (black arrowhead, C and D). Cell processes covered the perimeter of the aorta, and Z-lines are evident (arrowhead, D). Heart cardiomyocytes (cm) retract completely in a severely affected LIII (Segment A6) heart (asterisk, F), exposing naked fibrils of the heart ECM (arrowheads, F, G) The heart in (F) is flanked by fat cells (f). Scale 10 μm (A,C,F) and 1μm (B,D,E,G).

The structural consequences of reduced Talin function in cardiomyocytes was examined at higher resolution. We report here our observations on the aorta, as fragile heart chambers would fold and tear during histological processing. Ultrastructural study of the third instar (LIII) larval posterior aorta revealed vessels 30–40 μm in diameter, with a continuous circumference of myofibrils, rich in mitochondria and regularly spaced sarcomeres [37], (Fig 2A and 2B). In contrast, continuous depletion of Talin generated hearts and aortas of larger diameter (aortas of 50 to 80 μm diameter), with ultrastructurally normal myofibrils that encompassed a fraction of the heart diameter. Other domains of the circumference were comprised of cardiomyocyte cell processes lacking myofibril or sarcomere structure (Fig 2C–2E). Immunolabeled tissue suggested domains of the heart wall lack cellular structures (Fig 1D, 1F, 1H and 1J). Similarly, our EM analysis revealed that cardiomyocytes of severely affected hearts had retracted into thick cells, exposing naked ECM (Fig 2F and 2G). Serial section analysis shows that buried myofibril Z-lines did not terminate near the cell surface (data not shown), suggesting that costameres were not intact, as has been reported for body muscle [42].

**Fig 3:**
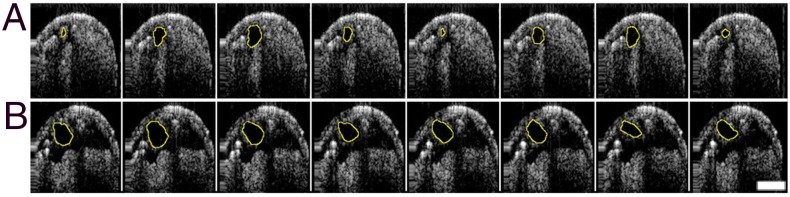
Fibrillation of heart contraction in Talin depleted hearts imaged by OCT. Cross-sections of the LIII heart in abdominal segment 6 selected at 100 msec intervals revealed complete contraction of the heart vessel in control larvae (A, *UAS-talinRNAi/+*). The heart perimeter is outlined. Hearts of LIII with Talin depleted during the first instar had dilated hearts with incomplete contractions (B, *UAS-TalinRNAi/+; Hand-Gal4*, *tubGal80* ^*ts*^ */+*).

The dilated heart phenotype of the Talin depleted heart was reminiscent of dilated vessel cardiomyopathy in mammals, suggesting that cardiac output would be similarly impaired. The cross-sectional profile of living *Drosophila* hearts can be visualised i*n vivo* from an Optical Coherence Tomography (OCT)-derived signal generated from heart wall motion [17, 43]. Wild type LIII hearts were capable of complete contraction, so that the cross-sectional area of the heart varied eightfold from systole to diastole (Fig 3A). In contrast, hearts of similarly staged larvae with continuous depletion of Talin had less than a twofold reduction in cross-sectional area during contraction (Fig 3B), suggestive of much lower cardiac output.

### Heart morphogenesis is more vulnerable than growth to reduced Talin function

At hatching, the *Drosophila* heart is not a mature contractile organ. In late embryogenesis, myofibrils are not present, and integrin is concentrated on the luminal face of the cardioblasts [37, 44]. After the heart begins to function, it must grow 4.7 fold in length before pupation (Fig 1I). During pupation, the heart remodels again to include the posterior aorta. Therefore cardiomyocytes are in constant change- they are either growing to accommodate the needs of a larger body, or modifying organ structure without addition of new cardiomyocytes. We have examined the functional requirements of Talin function during these stages by employing a temperature sensitive repressor of expression of Talin dsRNA [27, 45]. Ubiquitous expression of *GAL80* ^*ts*^ acts to sequester Gal4 protein, which is expressed in the heart under control of the Hand enhancer. At 29°C, Gal80^ts^ protein does not function, allowing Gal4 to activate expression of UAS-Talin dsRNA. Exposing growing larvae for differing times at 18 or 29°C allowed us to temporally restrict the period of Talin depletion, and then examine the consequences in adult hearts. Differing periods of Talin depletion further reduced the levels of immune-detectable Talin, only in the heart (panel 1C relative to 1A in S1 File). Normal levels of Talin expression were restored subsequent to a return to the permissive temperature for GAL80^ts^ function (panel B in S1 File).

**Fig 4:**
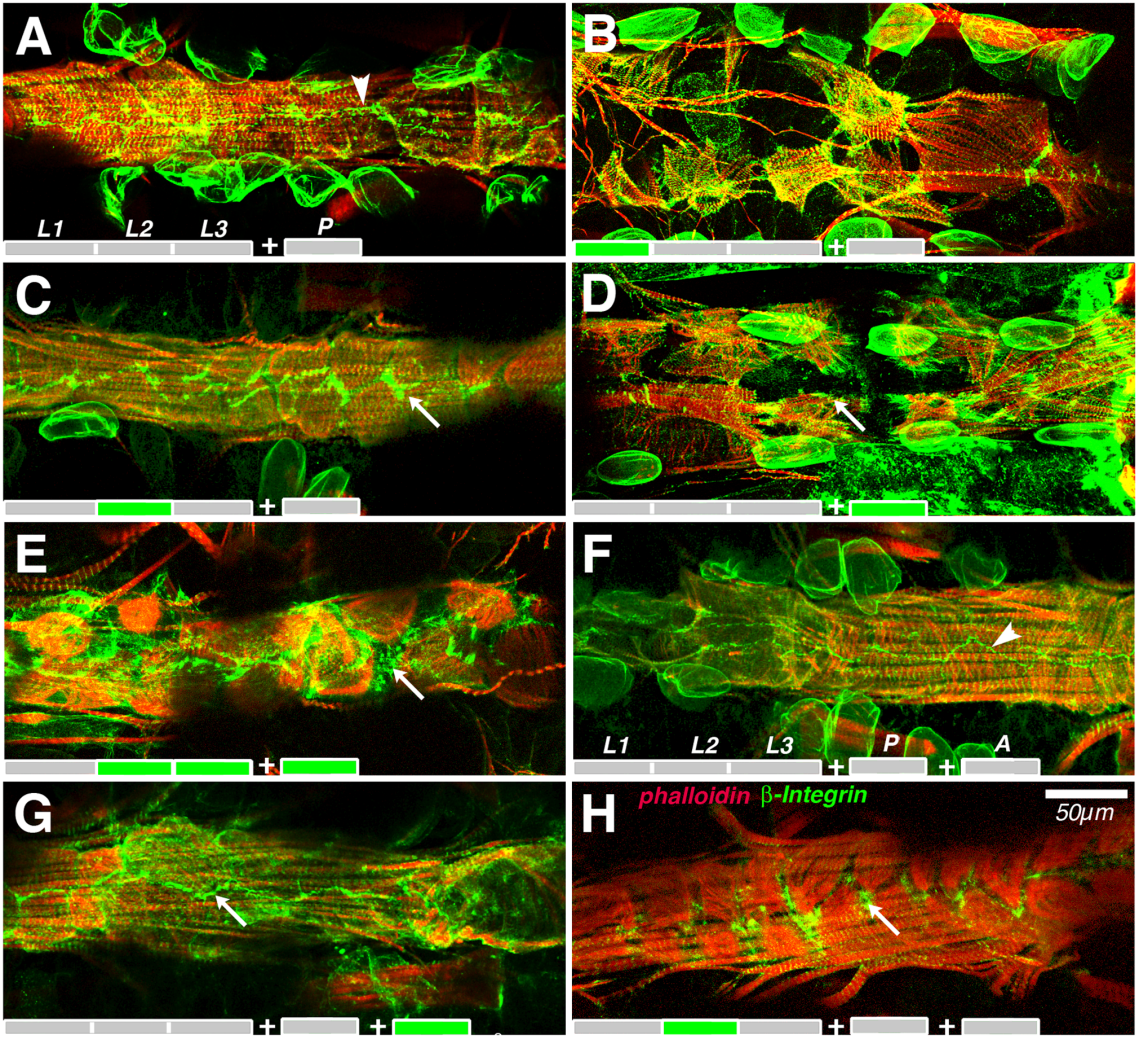
Cardiomyocytes do not re-connect after transient reduction of Talin function. In a wildtype adult, 6 days after eclosion, phalloidin labeled contractile fibres encompassed the entire heart, and integrin concentrated at cell insertions and the midline (arrowhead, A). This morphology was not altered after a further 6 weeks of aging (arrowhead, F). Talin was transiently depleted during larval, pupal or adult stages as shown by the coloured schema at the bottom of each panel, and stages labeled in (A). *UAS-talin RNAi; Hand-Gal4*, *tub- Gal80* ^*ts*^ flies were at 18°C during grey timeline, and Talin levels were normal., Hearts express the RNAi transgene, and deplete Talin levels at 29°C during the green timeline. Depletion during L1 created large gaps in myofibril coverage seen in the adult (B). Depletion during L2 created small gaps, filled with a broadened zone of integrin adhesion to the heart ECM (C). Depletion during pupal stages only generated an intermediate phenotype (D), comparable to depletion during L2, L3 and pupal stages combined (E). Depletion of Talin for 6 weeks of adulthood triggered small changes in the width of integrin labeled insertions (G). In contrast, gaps triggered in L2 persisted in 6 week old adults (H). Confocal projections of immunolabled adult heart dissections.

**Fig 5:**
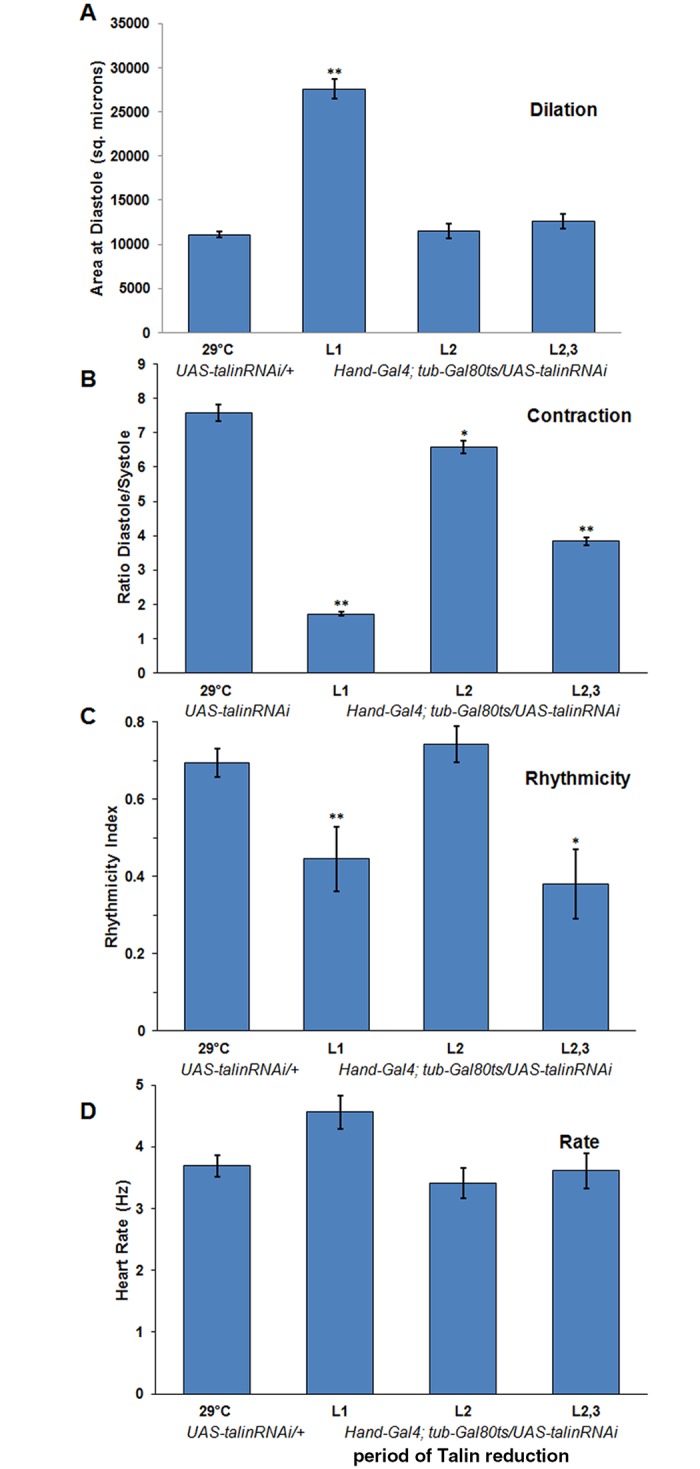
Talin reduction impairs cardiac rhythmicity but not heart rate. Cross-sectional area of the larval heart chamber (abdominal segment 6) was significantly dilated at diastole if Talin expression was suppressed during the first instar, but not other stages of larval growth (A, p<10^−5^, Mann-Whitney U test). The degree of contraction (ratio of diastolic to systolic cross-sectional area) was significantly reduced by depletion of Talin at any larval stage (B, L1- p<10^−5^; L2- p = 0.01; L2,3- p<10^−5^). The Rhythmicity Index (see Methods) was significantly reduced by depletion during L1 (p<0.005) and L2,3 (p<0.02). There were no significant differences in heart rate relative to control larvae for any treatment. OCT records of heart contraction were analysed for 6–20 *UAS-TalinRNAi/+; Hand-Gal4*, *tubGal80* ^*ts*^ */+* larvae for each temperature treatment and compared to 11 *UAS-talinRNAi/+* raised at 29°C. Period of RNAi expression on abscissa: first instar (L1), second instar (L2) and both second and third instar (L2,3). SEM indicated.

Unexpectedly, the period of greatest vulnerability to reduced Talin function was the first larval instar. In contrast to larvae raised entirely at 18°C (Fig 4A), larvae shifted to 29°C during the first instar had cardiomyocytes that were largely detached from one another, and not regularly placed along the length of the heart (Fig 4B). Similarly, these hearts were the most dilated, to more than twice the control cross-sectional area at diastole (Fig 5A). Resumption of Talin expression following first instar heat treatment could be delayed by the perdurance of Talin dsRNA, and the rate of Talin synthesis. Therefore we also looked at Talin depletion during the second and third instar, a period of tremendous growth in the heart. Depletion of Talin during second instar resulted in minor cardiomyocyte defects. Although the myocytes have enlarged during the larval growth, the myofibrils of adjacent myocytes no longer connect or align at narrow myocyte junctions, analogous to the intercalated disc of vertebrates (Fig 4C). This deficit shows no recovery subsequent to metamorphosis. Cardiomyocytes have tight apposition at the midline of controls raised at 18°C for 6 weeks (Fig 4F). Drosophila cardiomyocytes lack the uniform midline apposition if depleted in Talin only during second instar (Fig 4H). The gaps between myocytes are filled with expanded integrin adhesions, also producing an irregular alignment of cell appositions at the midline and a loss of myofibril alignment. Depletion of Talin during metamorphosis resulted in greater myofibril retraction, comparable to that seen with first instar depletion, but without the accompanying dilation of the vessel (Fig 4D and 4E). The midline apposition of cardiomyocytes was slightly widened by Talin depletion only during adulthood (Fig 4G), suggesting that turnover of Talin was reduced in post—growth myocytes. These data suggest that Talin is required at all pre-adult stages, but that the morphological consequences are more severe during remodelling rather than growth phases of development (summarised in S3 File). More significantly, early developmental defects in myocyte contacts and myofibril architecture are not repaired as the cardiomyocytes grow or remodel during metamorphosis.

How is heart physiology altered by the retraction of cardiomyocytes triggered by Talin depletion? OCT derived signals of beating hearts for all reduced Talin experiments were compared to parental genotypes (data is shown for *UAS-dsTalin/+*). In contrast to controls, only hearts with Talin depletion in the 1^st^ instar resulted in dilation of the heart (Fig 5A). However, Talin reduction at any larval stage was sufficient to reduce the contractility of the heart—measured as the ratio of the diastolic/systolic heart cross-sectional area (Fig 5B). The reduced contractility was much more severe if Talin levels were reduced during the 1^st^ instar, so that the heart appeared to fibrillate from asynchronous contraction of myocytes. This is reflected in the Rhythmicity Index, a correlation coefficient based upon analysis of the autocorrelation plot of the heart contraction cycle [35](Fig 5C). Again, the sensitive periods of early and late larval stages revealed the strongest reduction in rhythmicity. Although *Drosophila* larvae were acclimated to either 29°C or 18°C at the time of recording, there were no significant differences in heart rate when assessed at 24°C (Fig 5D).

In mammals, age related increase in hemodynamic load contributes to increased integrin expression [5]. Integrin expression also increases in aging *Drosophila* hearts, and the effects of aging on heart function can be phenocopied by overexpression of βPS-integrin in the heart [40]. This effect appears to require ILK, another IAC protein. Congruently, *Drosophila* with slightly reduced βPS-integrin function live longer [40]. Are these effects mediated by Talin, and what are the relative sensitivities to larval versus adult changes in Talin expression?

**Fig 6:**
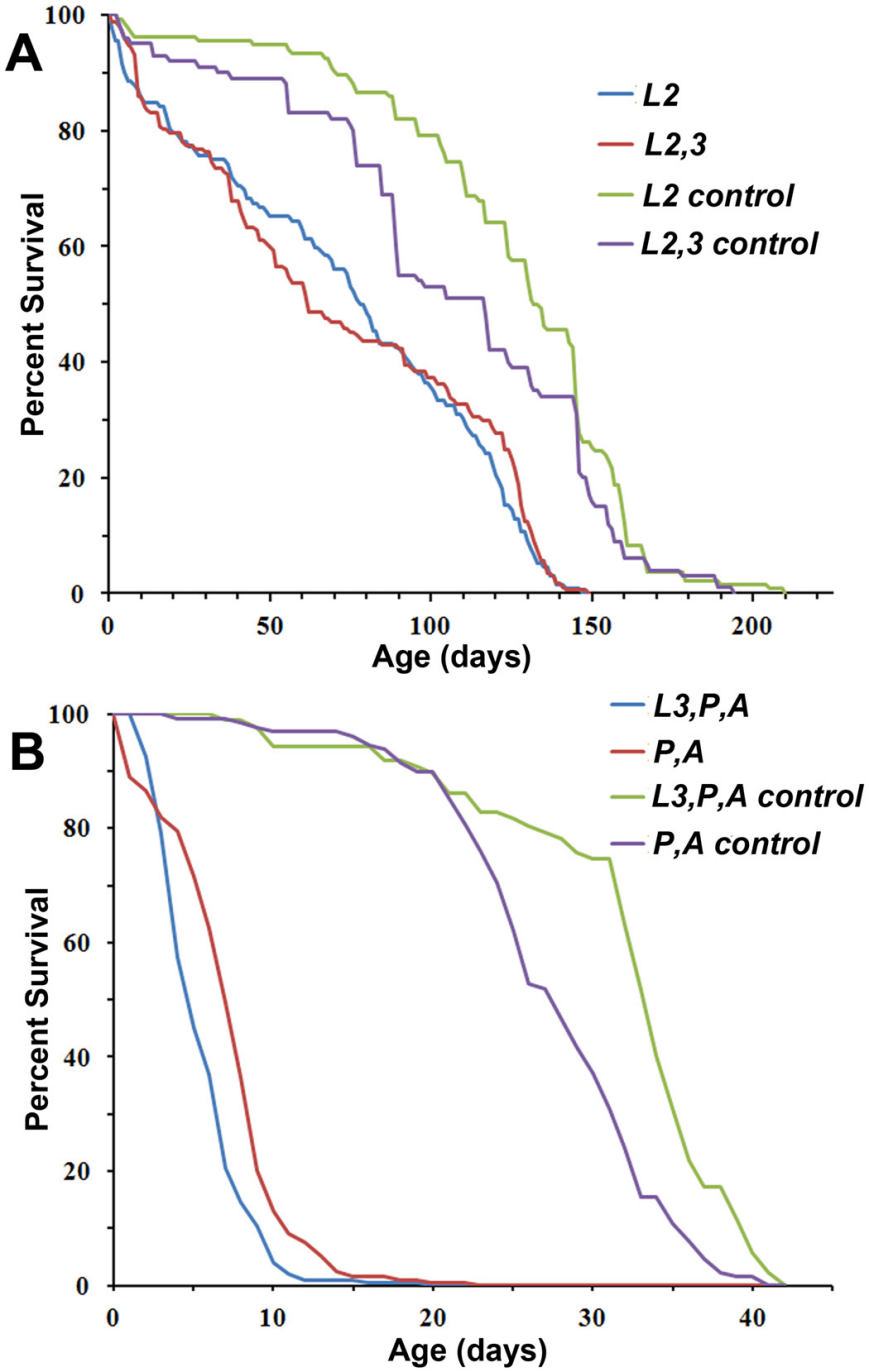
Lifespan is reduced by transient Talin reduction. Longevity of adults, subsequent to transient depletion of Talin during larval growth was tracked for 150 *UAS-Talin/+* or *UAS-talin RNAi; Hand-Gal4*, *tub- Gal80* ^*ts*^ adults for each treatment. Lifespan was dramatically shorter after Talin depletion in either L2 or in L2,3, and raised at 18°C (A), as well as raised with Talin depletion from L3 onwards or from pupation onwards at 29°C (B).

Employing standard aging protocols, we examined the longevity of male *Drosophila* subsequent to depletion during larval, pupal and adult life stages (Fig 6 and S4 File). Most larva with Talin depletion through the first instar died as pupae. Escapers lived a median age of 2 days (n = 27). Larva with Talin depletion during second or second and third instar only, and thereafter raised at 18°C passed 50% lethality at 81 and 64 days respectively, in contrast to controls with the same temperature profile, passing 50% lethality at 132 and 118 days respectively (Fig 6A). In contrast, *Drosophila* that developed with Talin depletion during third instar larva, pupal and adult stages, or only pupal and adult stages were short-lived at 29°C, reaching 50% lethality at 4 and 11 days, relative to controls (37 and 31 days, respectively, Fig 6B). Talin depletion during pupal and adult time dramatically reduced lifespan. These data suggest that the lack of regeneration after damage at any stage results in a permanent deficit that reduces lifespan.

## Discussion

In late embryogenesis, the *Drosophila* heart is a 4 micron diameter tube enclosed by 2 cardiomyocytes, attached at the dorsal and ventral midline with cadherin based cell junctions, and an integrin rich lumen [46, 47]. The larval cardiomyocytes are dominated by myofibrils that terminate in integrin rich insertions at the dorsal and ventral midline, without a cadherin rich domain. Therefore early heart development is marked by dramatic reorganisation of cell adhesion and polarity. For the remainder of a fly’s life, cardiomyocyte differentiation is remarkable for increase in cell size but not cell number, and for the pupal remodelling of posterior aorta myocytes into heart myocytes [48].

In this study, we examined the role of Talin production in the differentiation, growth and remodelling of cardiomyocytes. We verified the requirement of integrin function for cardiomyocyte adhesion [40], and note, like body wall muscle, that the insertions are Integrin-rich, and that the muscle costameres coincide with myocyte surface integrin adhesions. Normally, myofibrils of each cell are aligned end-to-end with myofibrils of the contralateral cardiomyocyte, suggesting that ECM linkages at the end of myofibrils are different from the rest of the cell surface, reminiscent of the mammalian intercalated disc. If levels of Talin production were reduced, cardiomyocyte insertion, particularly at points of myofibril termination were vulnerable to degeneration.

During first instar heart differentiation and pupal remodelling, cardiomyocytes were most susceptible to depletion of Talin, resulting in significant cell shrinkage. At less susceptible stages of heart growth, less cell shrinkage, but loss of myofibril apposition between cells results. The resulting degeneration of heart structure is likely due to the loss of adhesion caused by the depletion of Talin. This reflects the ongoing turnover of Talin and Integrin at adhesions, shown to be modulated by tension in *Drosophila* muscle [49]. Remarkably, restoration of normal Talin expression does not enable regeneration of myofibril length, inter-cardiomyocyte cell junctions or apposition of myofibril ends between myocytes at any larval or adult stage. Instead, the cardiomyocyte perimeter is marked by a broader band of integrin, suggestive of expanded adhesion to the heart ECM, and hence less direct transmission of tension between cardiomyocytes. Nevertheless, affected cardiomyocytes continue to grow as the larva grows, without restoring cell to cell apposition and alignment of myofibrils.

Heart contraction was reduced subsequent to Talin reduction at each larval stage, including during the second instar, when myocyte degeneration was minimal, but midline apposition of myofibrils was disrupted. Nevertheless, this disruption did not reduce the rhythmicity of second instar treated hearts. Heart dilation, rhythmicity and contraction were most affected by transient depletion of Talin during cardiomyocyte remodelling in the first instar, suggesting that synchronicity of cardiomyocyte contraction requires cell to cell contact, possibly along the ipsilateral domains of cardiomyocytes. This cell surface domain contains the costameres, where components of the IAC are implicated in tension signalling [8].

Myofibril stability may depend upon linkage to integrin adhesion at insertions, or at the costameres, as Talin depleted cardiomyocytes have fewer myofibrils. However muscle insertion structure was far more sensitive to the level of Talin than the structure of the costamere. Weakened costameres, observed in *Drosophila* mutants of muscle Trim32, are depleted of IAC proteins, including Talin, resulting in unbundling of myofibrils and muscle “wasting”[50]. Similarly, increased or decreased Integrin function in vertebrate heart muscle alters intercalated disc structure and cardiomyocyte contractility [51]. In *Drosophila* and vertebrates, integrin adhesion signalling is required for homeostasis of the contractile apparatus.

ECM is visible on the luminal and abluminal surfaces of cardiomyocytes (Fig 2). As heart diameter grows normally, new matrix must be deposited on both surfaces. Similarly, when cardiomyocytes retract, the remaining ECM likely stretches and expands as the heart vessel becomes dilated. In the Drosophila model, this dilation results in the deposition of a more elaborate network of Pericardin containing ECM fibrils. This process is analogous to mammalian Dilated Cardiomyopathy (DCM). DCM can be triggered by mutations in proteins that link the sarcomere to the ECM, such as IAC proteins vinculin and tintin [1, 52]. Expression of IAC proteins is elevated in cardiac hypertrophy [53]. Analysis of IAC gene function in genetic models such as *Drosophila* reveals the temporal dimension of the stability and remodeling of myofibrils. This study indicates myofibril stability requires ongoing Talin renewal, and that regeneration after perturbation is very limited. Further study of IAC function subsequent to changes in cardiac load in *Drosophila* cardiomyocytes should be instructive in revealing the signalling pathways activated in DCM.

## Supporting Information

S1 File Talin expression and depletion in the larval heart. In *UAS-TalinRNAi/+; Hand-Gal4*, *tubGal80* ^*ts*^ */+* larva raised at 18°C, Talin immunolabeling (green) is localised at the costameres of somatic muscle (*sm*) throughout the pericardial cells (*pc*) and concentrated at cardiomyocyte insertions at the midline (arrowheads, A). Larva raised at 29°C for the first instar only, and subsequently raised at 18°C have recovered comparable levels of Talin at the third instar (B). Hemocytes (*h*) frequently collect at the heart subsequent to cardiomyopathy. Larva raised at 29°C at all larval ages have lower levels of Talin immunolabeling in the pericardial cells and cardiomyocytes (C), while adjacent somatic muscle (*sm*) expresses high levels of Talin. Insets at right include the phalloidin labeling of myofibrils in red. Timeline is as shown in Fig 4. Calibration: 50 microns.
(TIF)

S2 File Larval heart rhythmicity. Analysis of OCT data for a *UAS-dsTalinRNA/+* larva at left, and a *UAS-TalinRNAi/+; HandGal4*, *tubGal80* ^*ts*^ */+* larva raised at 29°C during first instar, and then at 18°C, until filming during wandering phase of third instar. OCT M-mode plot of signal from a 30 micron window at the midline is plotted for 7 seconds (A). Correcting for dorsal or ventral movement, a de-trended plot of signal intensity was extracted (B). A Fourier transform was applied to the de-trended signal in order to quantify the heart beat frequency by locating the maximum peak in the frequency spectrum (C). The same de-trended signal (B) was analysed for the distribution of inter-peak intervals by calculating its Autocorrelation Function (ACF) to visualise the rhythmicity of heart contractions (D). The correlogram shape and values (D) indicate the Rhythmicity Index (RI) of the analysed data. The asterisk above the third peak (offset from 0 seconds, for which ACF is equal to 1) indicates the point used to determine the value of the RI.
(TIF)

S3 File Percentage of flies that died as pupae (as portion of total pupa) for the four treatments and four controls (n> 150 pupae/group). (DOC)

S4 File Phenotypes associated with Talin depletion at different stages. Alignment is the linearity of midline myofibril appositions. Gap is a measure of the distance across the midline to the closest contact between contralateral cardiomyocytes. Errors are ± S.D.
(PDF)
