# Supplementary figures and images for: Talin Is Required Continuously for Cardiomyocyte Remodeling during Heart Growth in Drosophila
Source: PLoS One. 2015 Jun 25;10(6):e0131238. doi: 10.1371/journal.pone.0131238 (PMC4482443; doi:10.1371/journal.pone.0131238)

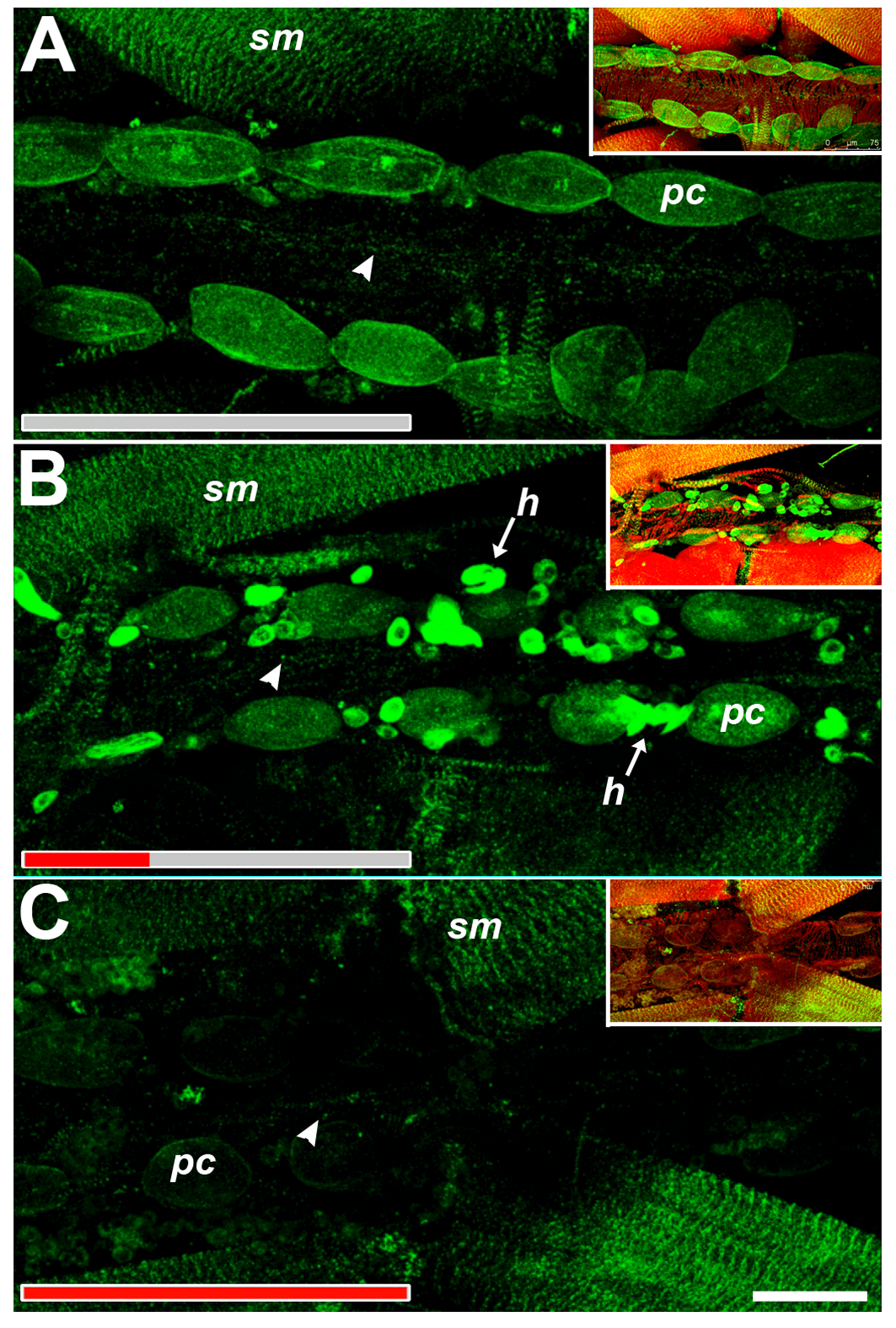

Supplement: S1 File — In UAS-TalinRNAi/+; Hand-Gal4, tubGal80 ts /+ larva raised at 18°C, Talin immunolabeling (green) is localised at the costameres of somatic muscle (sm) throughout the pericardial cells (pc) and concentrated at cardiomyocyte insertions at the midline (arrowheads, A). Larva raised at 29°C for the first instar only, and subsequently raised at 18°C have recovered comparable levels of Talin at the third instar (B). Hemocytes (h) frequently collect at the heart subsequent to cardiomyopathy. Larva raised at 29°C at all larval ages have lower levels of Talin immunolabeling in the pericardial cells and cardiomyocytes (C), while adjacent somatic muscle (sm) expresses high levels of Talin. Insets at right include the phalloidin labeling of myofibrils in red. Timeline is as shown in Fig 4. Calibration: 50 microns. (TIF) [file pone.0131238.s001.tif]

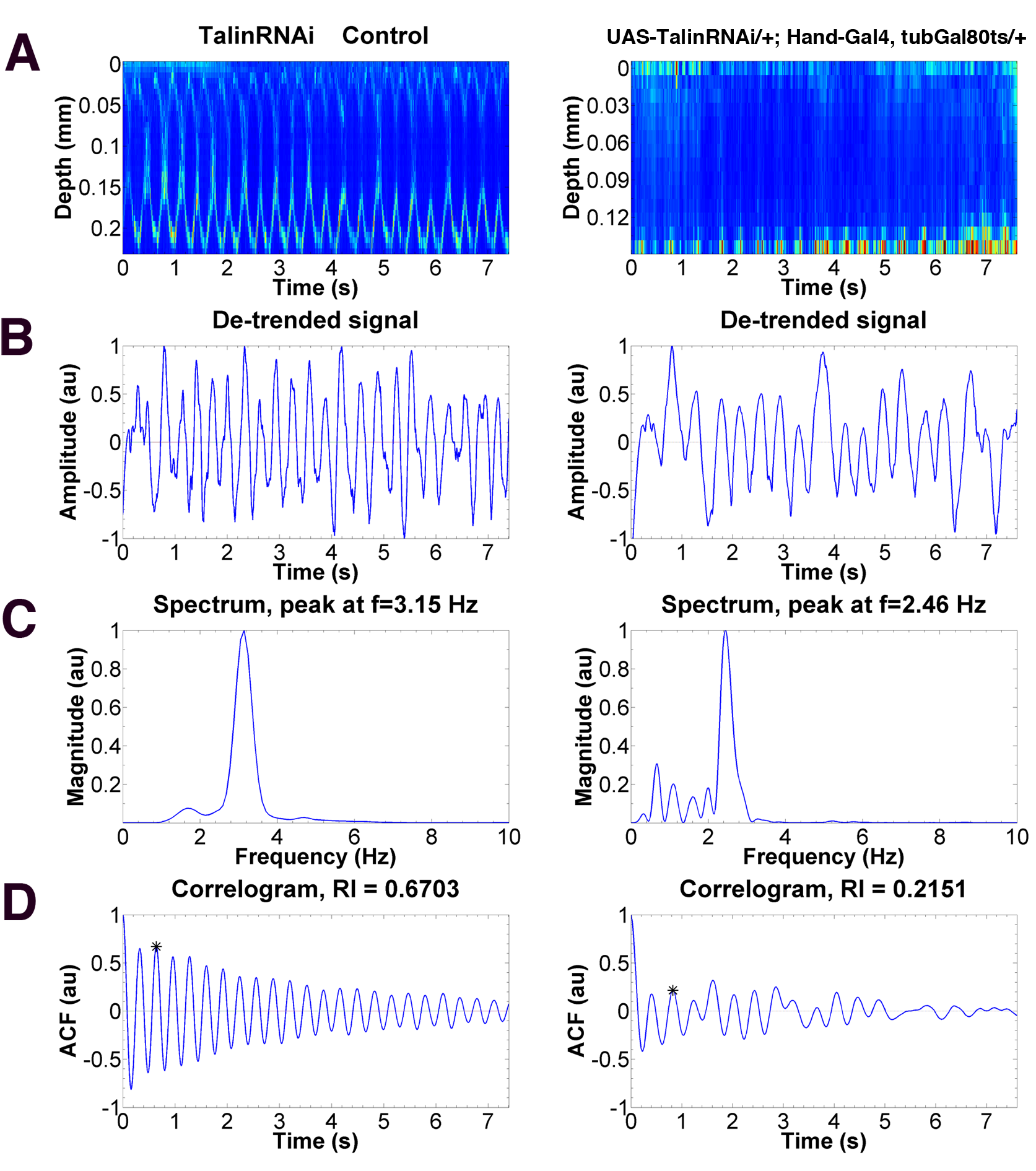

Supplement: S2 File — Analysis of OCT data for a UAS-dsTalinRNA/+ larva at left, and a UAS-TalinRNAi/+; HandGal4, tubGal80 ts /+ larva raised at 29°C during first instar, and then at 18°C, until filming during wandering phase of third instar. OCT M-mode plot of signal from a 30 micron window at the midline is plotted for 7 seconds (A). Correcting for dorsal or ventral movement, a de-trended plot of signal intensity was extracted (B). A Fourier transform was applied to the de-trended signal in order to quantify the heart beat frequency by locating the maximum peak in the frequency spectrum (C). The same de-trended signal (B) was analysed for the distribution of inter-peak intervals by calculating its Autocorrelation Function (ACF) to visualise the rhythmicity of heart contractions (D). The correlogram shape and values (D) indicate the Rhythmicity Index (RI) of the analysed data. The asterisk above the third peak (offset from 0 seconds, for which ACF is equal to 1) indicates the point used to determine the value of the RI. (TIF) [file pone.0131238.s002.tif]
