# Supplementary material for: Talin Is Required Continuously for Cardiomyocyte Remodeling during Heart Growth in Drosophila
Source: PLoS One. 2015 Jun 25;10(6):e0131238. doi: 10.1371/journal.pone.0131238 (PMC4482443; doi:10.1371/journal.pone.0131238)
Supplement: S3 File — (DOC) [file pone.0131238.s003.doc]

| **Depletion period (instar)** | **1^st^** | **2^nd^, 3^rd^** | **3^rd^, pupal** | **pupal** | **genotype** |
| --- | --- | --- | --- | --- | --- |
| **TalinRNAi** | 52.6 | 14.4 | 24.2 | 13.7 | *yw/+; UAS-dsTalinRNAi^VDRC 40399^/+; Hand-Gal4, tubGal80^ts^/+* |
| **Control** | 0.95 | 1.53 | 1.06 | 1.62 | *yw/+; UAS-dsTalinRNAi ^VDRC 40399^/+* |

**Supporting Table 1.** Percentage of flies that died as pupae (as portion of total pupa) for the four treatments and four controls (n> 150 pupae).
