# Supplementary material for: Talin Is Required Continuously for Cardiomyocyte Remodeling during Heart Growth in Drosophila
Source: PLoS One. 2015 Jun 25;10(6):e0131238. doi: 10.1371/journal.pone.0131238 (PMC4482443; doi:10.1371/journal.pone.0131238)
Supplement: S4 File — Alignment is the linearity of midline myofibril appositions. Gap is a measure of the distance across the midline to the closest contact between contralateral cardiomyocytes. Errors are ± S.D. (PDF) [file pone.0131238.s004.pdf]

Supporting Table S2.
Phenotypes associated with Talin depletion at different stages.

Depletion during:	**midline zone “intercalated disc”**		**medial orientation of myofibril**	**Contraction Ratio**	**Rhythmicity**	**Heart Rate, *Hz***
	**alignment**	**midline gap**				
**Control**	linear	< 5 µm	yes	7.58±2.08	0.69±0.08	3.69±0.45
**L1**	absent	10 - 50 µm	no	1.71±1.01	0.45±.020	4.56±0.67
**L2,3**	jagged	3 - 8 µm	yes	3.84±1.47	0.38±.023	3.61±0.75
**L1,2,3,Pupal**	jagged *or* absent	5 - 20 µm	no	*n.d.*	*n.d.*	*n.d.*
**Pupal**	jagged *or* absent	5- 50 µm	no	*n.d.*	*n.d.*	*n.d.*
**Adult (6 wk)**	linear	< 5 µm	yes	*n.d.*	*n.d.*	*n.d.*
